# Challenges and Lessons Learned from Multi-Level Multi-Component Interventions to Prevent and Reduce Childhood Obesity

**DOI:** 10.3390/ijerph16010030

**Published:** 2018-12-24

**Authors:** Joel Gittelsohn, Rachel Novotny, Angela Cristina Bizzotto Trude, Jean Butel, Bent Egberg Mikkelsen

**Affiliations:** 1 Center for Human Nutrition, International Health Department, Johns Hopkins Bloomberg School of Public Health, 615 N Wolfe St, Baltimore, MD 21205, USA; atrude1@jhu.edu; 2 Children’s Healthy Living Center of Excellence, College of Tropical Agriculture and Human Resources – University of Hawai‘i at Mānoa, Honolulu, HI 96822, USA; novotny@hawaii.edu (R.N.); jbutel@hawaii.edu (J.B.); 3 Department of Learning & Philosophy, Aalborg University, A.C. Meyers Vænge 15, DK-2450 Copenhagen SV, Denmark; bemi@learning.aau.dk

**Keywords:** childhood obesity, prevention, multilevel, community, evaluation

## Abstract

Multi-level multi-component (MLMC) strategies have been recommended to prevent and reduce childhood obesity, but results of such trials have been mixed. The present work discusses lessons learned from three recently completed MLMC interventions to inform future research and policy addressing childhood obesity. B’more Healthy Communities for Kids (BHCK), Children’s Healthy Living (CHL), and Health and Local Community (SoL) trials had distinct cultural contexts, global regions, and study designs, but intervened at multiple levels of the socioecological model with strategies that address multiple components of complex food and physical activity environments to prevent childhood obesity. We discuss four common themes: (i) How to engage with community partners and involve them in development of intervention and study design; (ii) build and maintain intervention intensity by creating mutual promotion and reinforcement of the intervention activities across the multiple levels and components; (iii) conduct process evaluation for monitoring, midcourse corrections, and to engage stakeholder groups; and (iv) sustaining MLMC interventions and its effect by developing enduring and systems focused collaborations. The paper expands on each of these themes with specific lessons learned and presents future directions for MLMC trials.

## 1. Introduction

Multi-level multi-component (MLMC) interventions are increasingly being tested as a means of addressing childhood obesity. MLMC interventions are thought to be more effective than single component interventions (e.g., schools only), because it is suggested that reaching children and their caregivers in different complementary settings, and with policy, will be more likely to impact the choices that people have (environmental change), what they choose to do (behavior) and ultimately, child obesity [1].

Previous reviews of the literature on multi-level multi-component obesity intervention programs using community-wide or population-based (or “whole-of-community”) approaches have reported positive but small effects on childhood obesity [2,3]. A systematic review of eight MLMC interventions found that seven reported a positive statistically significant effect on child obesity, and their meta-analysis reported a modest mean change in body mass index (BMI) z-score of −0.09 among intervention children compared to their comparison counterparts [2]. Another recent literature review found 14 studies that employed a MLMC strategy to prevent childhood obesity; of those, eight reported positive health and behavioral outcome results, five showed null results, and three demonstrated reductions on childhood obesity [3]. Therefore, despite some positive findings, previous MLMC trial impacts on preventing or reducing BMI among children have been small, with limited clinical and public health benefits [4].

In addition, several recent multi-level childhood obesity prevention programs have generated poor results. Four randomized, controlled trials that were part of the *Childhood Obesity Prevention and Treatment* (COPTR) consortium tested multi-level interventions to prevent obesity in preschoolers and treat obesity in children 7–13 years old in primary care, parks and recreational centers, households, and schools [5]. None of the COPTR studies found a significant improvement in child BMI [6,7,8,9]. Importantly, these studies did not intervene in the community food or physical activity environments.

Although the evidence base of MLMC interventions to address childhood obesity is growing, previous trials have shown mixed results and small effect sizes in child BMI, though still better than single component interventions [1]. Less work has been done on how to best impact low income diverse populations, and there is a need to consistently incorporate environmental interventions into systems to sustain the interventions and create fundamental community change [10]. A great challenge in MLMC interventions, and possibly why effects seen have not been stronger, is that in such complex trials researchers do not know at what levels or how to intervene, or how to best monitor and evaluate these complex interventions. Essentially, MLMC interventions represent an attempt to intervene in complex systems by addressing multiple components of the system simultaneously. Systems science approaches may be especially relevant in this case to help us understand the complex relationships between and within socioecological levels [11,12], but also raise several important questions, such as: Are some socioecological levels or systems components complementary with others? Are some combinations of intervention strategies better than others? Is there an ideal sequence of program implementation? Are there interactions and feedback loops? What is the best way to ensure adequate intensity and exposure (i.e., dose and duration) in these complex interventions to affect behavior change? Therefore, there is a great need to consider lessons learned from multiple MLMC intervention trials, and to study implementation experiences carefully to inform future work.

The aim of this paper is to describe key lessons learned from three recently completed MLMC trials through the lens of systems science, focusing on: (1) Study design and community engagement, (2) building and maintaining intervention intensity, and (3) sustaining MLMC interventions and impact.

## 2. Methods

A case study approach was used, guided by program theory. All three MLMC interventions were grounded in ecological systems theory and applied the social ecological model to the design and implementation of the interventions. Each of the three cases (BHCK, CHL, SoL) have published multiple papers describing design [13,14], formative research [15,16], process evaluation [17,18], and impact findings [19,20,21]. The principal investigators (PIs) of the three interventions have presented and discussed their findings and experiences through a series of five consecutive ISBNPA (International Society of Behavioral Nutrition and Physical Activity) symposia. The analytical approach has been developed in preparation for these symposia and this paper by the PIs, and has taken the form of extended discussions and the preparation of cross-case matrices. The overall aim has been to identify the strengths and limitations of each MLMC trial, and to identify the approaches, tools and components that need to be deployed to increase the impact on childhood obesity and to secure the long-term sustainability of the program. We have identified general principles that could enhance the intervention effect and factors that are dependent on the local socioeconomic and cultural context of each of the three cases. Thus, the information presented in this paper emerges both from the published literature and from the experiences of the principal investigators (JG, RN, BEM).

B’more Healthy Communities for Kids (BHCK) was implemented at the policy, wholesaler, small food source, recreation center and social media levels to modify the food environment, and provide nutrition education and skills and reduce child obesity in 30 low-income neighborhoods in Baltimore City, Maryland, U.S. [14]. In contrast with the other two MLMC trials, BHCK did not implement strategies to promote physical activity and focused on education and modifying aspects of the out-of-school food environment for children 9–15 years old. BHCK was implemented in two waves lasting 6 and 8 months, respectively. The intervention demonstrated improvements in availability of healthier foods and beverages at multiple levels [21], and showed increased purchasing of healthy foods and beverages among intervention youth (β = 1.4; 95% CI: 0.1; 2.8), and a decrease of 3.9% kcal from sweet snacks in intervention adolescents (13–15 years old) versus comparison adolescents (β = −3.5; 95% CI: −7.76; −0.05) [22]. On the other hand, no impact was seen in sales of healthier foods in community food sources, nor in terms of improvements in children’s energy, fat or fruit and vegetable intake. BHCK was conducted according to the guidelines laid down in the Declaration of Helsinki and all procedures involving human subjects were approved by the Johns Hopkins Bloomberg School of Public Health Institutional Review Board (IRB #00004203).

The Children’s Healthy Living program for remote underserved minority populations of the Pacific (CHL) developed a community-driven multi-level multi-jurisdiction intervention that aimed to build social/cultural, physical/built, and political/economic environments to promote active play and intake of healthy food to prevent young child obesity in the Pacific Region. CHL implemented 19 activities focusing on preschool wellness and food environment policies, community environment change, promoting the CHL message and capacity building through training the trainer in 27 communities in 5 jurisdictions (countries/ states/ territories/commonwealths of the U.S.) [13,23]. The 26-month community randomized trial among 2–8 year-olds demonstrated greater improvements among 9 intervention communities compared to 9 matched comparison communities in overweight and obesity prevalence (effect size [d] = −3.95%; 95% CI, −7.47% to −0.43%), waist circumference (d = −0.71 cm; 95% CI, −1.37 to −0.05 cm), and acanthosis nigricans prevalence (d = −2.28%; 95% CI, −2.77% to −1.57%). Age and sex subgroup analysis revealed greater difference among intervention communities in acanthosis nigricans prevalence in the group aged from 2 to 5 years (−3.99%) compared to the group aged from 6 to 8 years (−3.40%), and the interaction was significant (d = 0.59%, *p* < 0.001), as well as a smaller difference in the group aged 2 to 5 years (−0.10%) compared to the group aged 6 to 8 years (−1.07%) in screen time (d = −0.97 hour per day, *p* = 0.01) [19]. However, none of the behavioral variables (fruit and vegetable consumption, physical activity, sugar-sweetened beverage and water consumption, sleep and screen time) showed significant overall differences between intervention and control communities. The Children’s Healthy Living program was approved by the University of Hawaii at Manoa Committee on Human Studies number 18915.

The SoL (Health and Local Community) program was a multilevel intervention targeting food/nutrition behavior and sedentary lifestyle among children aged 3–8 years and their families. SoL was implemented for 24 months in three neighborhoods of the community of Bornholm and as a matched control in three neighborhoods of the community of Odsherred, located on the island of Zealand off the coast of Denmark. Interventions were developed and implemented in childcare centers, schools and supermarkets, as well as in local mass media and social media. [16]. The supermarket intervention component improved consumers’ perception and attitudes towards healthier snacks, and increased sales of vegetables [20]. Effects on the individual behavioral level were modest, partly explained by the limited length of the intervention. The consumption of whole grain foods increased by 25%. Consumption of sugar-sweetened beverages among children decreased at the first follow up. However, the intervention did not demonstrate reduction in waist or BMI measures among children. The SoL (Health and Local Community) program was approved by the Institutional Review Board, the Ethical Committee of the Capital Region with the Project Identification Code: 3-2013-036.

## 3. Findings

**Figure 1:**
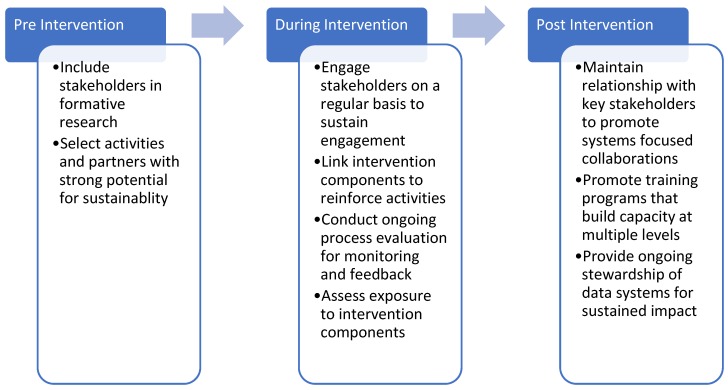
Summary of lessons learned by three multi-level multi-component (MLMC) community-based childhood obesity prevention studies.

Figure 1 summarizes the main lessons learned, divided into three groupings by program stage.

### 3.1. Design and Engagement (Pre-Intervention)

#### 3.1.1. Lesson #1: Use Early Stage Formative Research Including Stakeholder Analysis to Select Intervention Levels and Components

MLMC community interventions need to be developed in close cooperation with diverse stakeholders and with high sensitivity to socioeconomic and cultural contexts. Emphasis on this stage was crucial to secure successful program implementation and long term sustainability of interventions in all three studies. Stakeholder theory is a useful foundation that can guide the process of identifying the relevant stakeholders and their resources. It can also identify other resources available in the community as well as potential settings where the program can be developed and implemented. A stakeholder approach acknowledges the importance of a detailed mapping of stakeholders, which are the ones that will not only potentially benefit, but also will be important partners in the subsequent development, implementation and sustainability of the program [13,24].

Investing time in developing an understanding of the stakeholders, their needs and their resources, is an important activity in the pre-program phase, tailoring the program for the particular community and informing the formative process of systems research of the program–the part that will help to explain why a program works, which components are working and those that are working less well or not working. Each of the three MLMC programs selected and engaged stakeholders in different ways. For BHCK, the MLMC study was preceded by more than a decade of work with many of the same stakeholders, and so the work mainly involved re-establishing and expanding existing relationships [25,26,27,28,29]. In CHL, a formal process of stakeholder identification and engagement was developed, also building on work and relationships developed over more than a decade [15]. For SoL, the stakeholder process took place over two years and included meetings, visits, excursions and mail correspondence. The process included drafts and suggestions from all stakeholders on possible intervention components, suggestions for intervention design and proposals for practical ways to maximize stakeholder involvement during the course of the program. 

#### 3.1.2. Lesson #2: Select Intervention Activities and Partners that Have Strong Potential for Sustainability (e.g., Already Doing Similar Work, Capacity Building)

Intervention components for programs will often be suggested by researchers according to their knowledge about their effectiveness in other contexts or based on researchers’ theoretical insights. Furthermore, researchers tend to prefer program designs that favor easy collection of data. However, the components and program design also need to make sense to the local community’s residents. Insights from the three trials suggest that it is important to create conditions where negotiations between researchers, community leaders and representatives of local residents can take place. Locals often tend to have preferences for the idea of building on initiatives and actions that already exist in some form since they are well known to community members and are known to be socially acceptable. An important role for researchers is, therefore, to carefully identify such initiatives and to create forums where discussions can take place on how to further develop such actions and initiatives. Such forums can take many forms, including workshops, action group meetings, future scenario seminars and other participatory meeting forms. Discussions need to be able to identify how existing resources can create value for residents. The use of such strategies enhances the potential for long-term program sustainability.

For BHCK, these negotiations took place through initial community workshops and via a series of ongoing policy working group meetings [30,31]. This approach led to the identification of college and high school youth leaders, who both delivered the intervention and served as role models for younger youth. Following the ANGELO Framework and positive deviance theory, CHL intervention activities built on local initiatives, identified and promoted community members who modeled healthy behaviors and focused on activities determined to be important and achievable by the community [13]. Community meetings were held where facilitated discussions were held and decisions made. The SoL program worked with two municipalities and negotiated the terms, conditions and concepts of the program. All villages and potential candidates for settings were paid a visit in order to assess the local stakeholders’ willingness and commitment to play an active part in the intervention delivery, and to agree on the scope of the different intervention components and plans for coordinating activities across the participating villages. 

### 3.2. Building and Maintaining Intervention Intensity (During Intervention Implementation)

#### 3.2.1. Lesson #3: Use Mid-Stage Process Research and Stakeholder Management and Coalition Development to Sustain Engagement

Interventions seldom occur in isolation and often exist in a very dynamic environment where other policies, programs and initiatives coexist in the same community, and can change over time. This can result in needs for changes and modifications during the implementation and course of MLMC programs. Organizing dynamic stakeholder management with some type of local action group is a useful way to sustain engagement, and was an approach used in all three MLMC trials. In the BHCK trial, this was accomplished through regular meetings of the policy working group [31]. Flexibility was important: when local city councilpersons requested simulation modeling to support food environment improvement legislation, the BHCK team was able to provide model findings and gave supportive testimony [32]. In CHL, quarterly meetings with local advisory councils involved discussions of intervention activities and other similar activities being conducted in communities. Information from these meetings was shared with the CHL-wide intervention team and served as guideposts to direct intervention activities. For example, several communities were focusing on policies to reduce sugar-sweetened beverage consumption. In response, CHL used data collected at baseline to provide support for the proposed policies. For SoL, continued stakeholder support was secured by regular meetings in the local action groups and via meetings with local setting stakeholders (professionals at school, kindergartens and food stores). In addition, regular interactions with media actors served to reinforce the stakeholder relationships.

#### 3.2.2. Lesson #4: Create Linkages between Intervention Components Based on Complementarity, Mutual Promotion and Mutual Reinforcement

In MLMC interventions, linkages between intervention levels and components must be carefully considered in both design and implementation phases to achieve adequate intensity. Intervention components should not be siloed and separate. We have identified three types of linkages between intervention components: complementarity, mutual promotion and mutual reinforcement.

Complementary interventions may address different groups within a target population. For example, in the SoL intervention, different components addressed children (school), adults (supermarkets) and policymakers (local action groups) [33]. Getting different groups involved in an intervention means that there will be enhanced opportunities for social support [33].

Mutual promotion in MLMC interventions means that the different intervention components refer to and support each other. As an example, the CHL program used newsletters and other media to link together different intervention components. These newsletters described different intervention activities focused on a particular topic (e.g., water consumption) and identified role models in each setting that performed the targeted behaviors. Water consumption was also discussed as a substitute for sugar-sweetened beverages, which was role modeled in various settings, and which was included in preschool wellness policies.

Mutual reinforcement in MLMC interventions focuses on building positive feedback loops to support positive or reduce negative behaviors. An example of this comes from the BHCK trial, where we worked with wholesalers to increase access to healthier foods and beverages for corner stores, who in turn stocked these foods, creating improved access for local consumers [21]. These consumers then increased demand for these products, creating a sustainable, positive supply–demand feedback loop. Understanding these linkages, and the form of the linkages (e.g., interactions and feedback loops) is part of taking a systems approach to MLMC intervention design.

#### 3.2.3. Lesson #5: Conduct Process Evaluation for Monitoring and Feedback

Conducting a thorough process evaluation is now an accepted and common practice in behavioral intervention trials. There is less agreement and fewer actual practice guidelines on the best ways to use these data. Should process data be used for ongoing monitoring and feedback and delivery of interventions? How often should this be done? How should this information be used to modify or maintain interventions at current levels?

**Figure 2:**
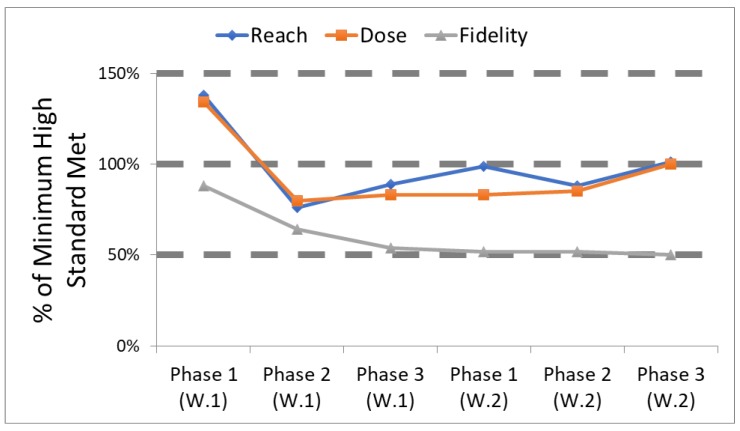
Tracking how well process evaluation standards were met for the youth leader component in B’More Healthy Communities for Kids. High (100%), medium (50%–99%) or low (<50%) refers to standards set a priori.

All three MLMC intervention trials conducted some form of process evaluation, with ongoing feedback. Perhaps the most formal version was employed by BHCK [18]. In BHCK, each intervention component had multiple measures for reach, dose delivered and fidelity [31,34,35,36,37]. Each of these measures had a minimum high standard for delivery, based on previous work, the literature, and/or specific resource constraints. Table 1 presents the standards set for delivery of the youth leader component of the BHCK intervention. For ease in communicating findings to our intervention team, we classified the degree to which each minimum high standard was met, where <50% of the high standard was viewed as low, 50%–99% was viewed as medium, and 100% of the standards set a priori was considered high. Every two months, roughly timed to coincide with the end of an intervention phase, the intervention team met and reviewed how well each standard was being met, and developed plans for improvement if reach, dose delivered and/or fidelity was below the minimum high standard (Figure 2). These regular meetings allowed the team to strengthen its efforts to improve specific aspects of the intervention, and over time led to enhanced program delivery [18].

The CHL intervention developed a monthly process reporting template. Each month, jurisdictions were required to record actions being taken to plan, facilitate, and delivery each of the 19 intervention activities. Reports included where the activity took place, how many participated, the progress of the activity, and their next steps. The qualitative reports were purposefully broad to capture community tailoring of activities. The report template was based on the RE-AIM (reach, efficacy, adoption, implementation, and maintenance) framework, prompting sites to report reach (number of expected and actual participants), implementation (types and number of intervention activities), Adoption (number of partners), and maintenance (next steps). Reporting monthly on intervention activities allowed for the coordinating center to monitor activities, identify strengths and areas for intervention improvement, and leverage ideas for use in other intervention communities, as well as track the intervention process [38]. The monthly reports were also quantified to provide information on the implementation process of the complex intervention.

#### 3.2.4. Lesson #6: Assess Exposure to Explain Findings

The three MLMC interventions all assessed exposure to the intervention, but in different ways. Exposure measures allow researchers to understand how well a program reached its intended audience from the participants’ perception of their personal exposure and the extent to which they actively engaged with the research activities and materials. BHCK collected data and calculated intervention-component specific exposure scores immediately post-intervention. CHL created activity dose scores post-intervention, while SoL constructed awareness scores conducted at one and two years post-intervention.

BHCK investigators developed both an overall exposure score and component-specific exposure sub-scores, that provide context to the impact analysis and can also be used to infer impacts [39]. BHCK findings suggest that high adult caregiver exposure to the BHCK social media component was associated with a threefold increase in fruit and vegetable consumption [40]. CHL created a dose score that reflected the number of activities conducted during the intervention, the estimated potential effectiveness of the activity, and the number of participants in each activity. Higher community intervention dose was correlated with a reduced screen time [41]. Findings also suggest that the combination of activities in the intervention package, such as combination of preschool policies to limit screen time, social marketing campaigns promoting limiting screen time and teacher trainings on activities to replace screen time, may have been related to the decrease in screen time.

### 3.3. Sustaining MLMC Interventions (Post-Intervention Implementation)

#### 3.3.1. Lesson #7: Form Enduring, Systems Focused Collaborations that Plan for Sustainability from the Beginning

To implement complex community interventions, a “place-based” organizing framework involving collaboration among community-based partners has been recommended [42], and the literature indicates that the development of community-based coalitions has the potential to lead to better health outcomes [43,44]. Lack of local support, leadership, or resources can create significant challenges in implementing interventions using the social-ecological approach [45]. The implementation of complex interventions that utilize the social-ecological approach requires building collaborative capacity to take collective action [46]. All three of the MLMC trails established coalitions among large organizations, and policy focused coalitions.

Large organizations included local health departments, parks and recreation departments, government offices of planning, and food sources in the BHCK intervention. As an example, BHCK worked to create sustainable, multi-level supply and demand loops between wholesalers, corner stores and customers. CHL focused on land grant colleges as the foundation for large organization coalitions. SoL partnered with local municipal health departments, whereas BHCK formed a policy work group to support specific city policies and to determine strategies to sustain intervention components within. CHL’s policy focused coalitions varied with jurisdiction. For example, several jurisdictions formed coalitions with the non-communicable disease task forces and one worked with an obesity prevention task force. The participation and forming of multilevel coalitions served a critical role to engage partners in the implementation of the intervention and to adopt activities to ensure sustainability.

#### 3.3.2. Lesson #8: Require Ongoing Training Programs at Multiple Levels

For MLMC interventions to be successful, capacity development is needed and, in particular, the development of skills to enact the desired change [4]. The three MLMC interventions used a multi-level approach to skills development. Three levels of training were addressed in the interventions (program participants, local practitioners, and early career researchers). These levels of training were integrated into the intervention to develop skills and address different audiences. 

The BHCK intervention trained community members along with recreation center staff (local practitioners) in the implementation of program activities as well as engaged youth to be peer mentors and train other youth to be mentors. To build research capacity, simulation modeling was incorporated into the policy work group. 

CHL embraced capacity development as the region that lacked childhood obesity training programs. In addition to a degree training program, to promote sustainability and develop capacity in the region, the CHL intervention used the train-the-trainer approach. Due to the age of program participants (2–8 years old), CHL focused on training parents, caregivers, older siblings and community role models to conduct activities related to the CHL target behaviors. For example, role model training developed communication skills among participants to enable them to be effective community messengers and advocates. This approach was applied to teachers and community practitioners through hands-on training that they could then teach to young children. For example, workshops were conducted with preschool teachers on ways to engage young children in physical activity and promote healthy eating at mealtimes. Another example involved training HeadStart teachers to talk with parents about child BMI. The CHL program also provided scholarships to college students to develop a network of childhood obesity professionals in these jurisdictions and build capacity at land grant colleges in the jurisdictions. Training in research methodology and data collection was provided to CHL jurisdiction staff, further developing a network of childhood obesity professionals in the region.

The SoL program sought to change the perspective of food by the community. They looked to expand the view of food from being a narrowly defined as what one “should or should not” eat to a broader understanding of the role of food in promotion of better health. 

#### 3.3.3. Lesson #9: Ongoing Stewardship of Data Systems is Required for Sustainable Impacts

Making use of collected data is important for generating new knowledge in science and also for community use. All three MLMC trials have developed different strategies for making data available. BHCK is using project data to develop systems science simulation models in support of city policies and programs [30]. Specific analyses are made available to different city agencies at their request (including Recreation and Parks, Health departments). In CHL, substantial in-depth community reports were provided to each participating community. CHL maintains a website as a source of community and scientific information, and as a portal to request data for research or other uses (chl-pacific.org). Tools, such as the Pacific Tracker for diet analysis containing Pacific foods, are also available on the website. For SoL, data management was delegated to a participating research center. The data sets have been used for a number of customized analyses made for project beneficiaries, including a wide range of doctoral and master students, as well as trainees, that have been credited to the program. 

## 4. Discussion

The interaction between PIs representing three diverse MLMC intervention trials has been very fruitful. All three trials identified similar lessons learned, but have addressed each general lesson in different ways. Importantly, the PIs have been interacting and discussing their projects in a comparative manner for over five years, generally since the time each project was implementing its intervention delivery work. Clearly there has been a learning process and exchange of strategies due to this interaction over the years.

Many of our lessons learned, in terms of community collaboration at multiple stages (pre-, during, and post-intervention), have been identified in other MLMC obesity intervention trials, and are representative of community based participatory research [47]. The Romp and Chomp trial emphasized capacity building heavily, including (i) a commitment to long-term efforts and to maintain collaborations at all levels; (ii) ongoing intervention activities to foster and maintain networks and partnerships; (iii) activities with a focus on leadership skills within the implementation team; (iv) and ongoing evaluation rooted in a theoretical framework [48]. Certainly, strategies for collaboration and building engagement are common to most community-based interventions, whether or not they are multi-level. 

Despite promising early evidence of the effectiveness of MLMCs to reduce and prevent childhood obesity, effects on preventing or reducing BMI among children have been small, with limited clinical and public health significance [45]. With multiple intervention strategies occurring simultaneously in different community settings, MLMC interventions make it difficult to attribute the effect of the trial to specific intervention strategies, or even to the intervention as a whole due to the presence of community-level confounding variables [46]. Furthermore, community-based intervention trials often assume that the intervention protocol was implemented according to initial standards, but programs are often adapted to the reality and the needs of the community, or may not reach their intended target population, which may explain reduced treatment effects [4]. One of the main findings of our group is the need for attention to intervention frequency and intensity, how to define it, how to set it and how to monitor or adjust for it. MLMC trials are complex, with many components which require substantial resources to implement, and close attention to develop strategies to employ simultaneous and mutually supportive interventions. In addition, it may be beneficial to look beyond the obesity prevention and control literature. A common factor drawn upon strategies to prevent or control other epidemics (e.g., tobacco, HIV, drunk driving) is the successful social mobilization and shift in social norms [49]. Strategies that facilitate the organization of advocacy groups and grassroots movements to change community attitudes and perceptions related to obesity-related behaviors may influence upstream interventions, including regulatory and economic approaches (e.g., taxes, marketing bans, universal access to clean water and physical activity opportunities) [50].

Our collaborative work has identified several limitations of MLMC interventions. One of the key first limitations we have found is a lack of common terminology. The term “multi-level, multi-component” (MLMC) interventions is our invention, and time will tell if it is acceptable to the broader public health research community. Our terminology/concepts for improving intervention intensity in MLMC programs (mutually supportive, referential, etc.) is also new with our collaboration, and reflects an important dimension of planning and practice. An additional limitation lies in the variety of approaches for assessing and using process information. Our approaches ranged from minimal (SoL) to intensive (BHCK) to broad (CHL). More detailed process evaluation potentially tells you more, and offers greater opportunities for adjustment, but is also very costly. We have yet to arrive at a common agreed upon set of standards for process evaluation of these complex studies.

A final limitation of this paper is that the three MLMC child obesity intervention trials referenced focused on children in different age groups (BHCK, 9–15 years; CHL, 2–8 years; SoL, 3–8 years). It is quite possible that different combinations of interventions would be more successful depending on the age of the children, and that other important lessons would emerge.

What are some promising future directions for MLMC intervention work? Other investigators have taken data and lessons learned from successful MLMC childhood obesity prevention interventions such as Shape Up Somerville [51] and Romp and Chomp [52] to develop, test, and refine MLMC strategies using systems science approaches, such as participatory model building exercises, social network analyses [53], and agent-based models [11]. The international collaboration known as COMPACT (Childhood Obesity Modeling for Prevention and Community Transformation) is currently using system science principles to leverage community-based childhood obesity interventions [11]. 

## 5. Conclusions

MLMC interventions represent a promising, albeit complex, approach for addressing the childhood obesity epidemic. While it seems apparent that a multifactorial problem like obesity will require multifactorial solution, surprisingly little synthesis of the literature has taken place. This paper addresses these limitations by presenting the collaborative work of three investigators who have undertaken separate MLMC interventions, each in diverse settings. Over a series of five years, the investigators have shared their ongoing experiences through a series of joint symposia and associated discussions. The lessons learned from these shared experiences have been divided into planning, implementation and sustainability phases in this paper. Some of the important key lessons learned for these interventions include: Identifying partners based on potential sustainability, the importance of developing mutually referential and supportive interventions, regular monitoring and feedback, and the importance of exposure assessment. We hope this work provides a useful framework for future investigators working on complex interventions.

## Figures and Tables

**Table 1:** Process evaluation standards for the BHCK recreation center component.

Recreation Center/Peer-leader Component	Set Standard (# for optimal)
Reach	
# of children per session (10–14)	10+
Dose Delivered	
# handouts distributed/session	12+
# giveaways distributed/session	12+
# food sampled per session	12+
# types of recipes distributed (phase 2)	3+
# posts featuring youth-leaders	3+
Fidelity	
# of YL attending each session	5+
# of visits to after-school center/ week	3+
# of YL in corner store session/phase	10+
# of posters up at after-school center/phase	3+
# of post made by YL per phase	3+
% of YL attendance to trainings	90%
# of YL actively participating	10

Dose delivered = units of intervention materials/activities (e.g., nutrition sessions, posters, flyers) provided by BHCK interventionists. Fidelity = quality of intervention component implementation, based on reactions to or engagement with the program. BHCK = B’More Healthy Communities for Kids. YL = youth leader.
